# Deep Learning-Based Prediction of Fish Freshness and Purchasability Using Multi-Angle Image Data

**DOI:** 10.3390/foods15010068

**Published:** 2025-12-25

**Authors:** Sakhi Mohammad Hamidy, Yusuf Kuvvetli, Yetkin Sakarya, Serya Tülin Özkütük, Yesim Özoğul

**Affiliations:** 1 Department of Industrial Engineering, İstanbul Arel University, 34537 İstanbul, Türkiye; 2 Department of Industrial Engineering, Cukurova University, 01330 Adana, Türkiye; 3 Department of Seafood Processing Technology, Cukurova University, 01330 Adana, Türkiye

**Keywords:** fish freshness, transfer learning, deep learning, fish quality analysis, CIELab color analysis

## Abstract

This study aims to predict the freshness of sea bass (*Dicentrarchus labrax*) using deep learning models based on image data. For this purpose, 10 fish were monitored daily from the day of purchase until three days after spoilage, with multi-angle imaging (eight distinct perspectives per fish, both with and without background) and corresponding quality analyses. A total of 22 quality parameters—10 categorical (sensory-based) and 12 numerical (color-based)—were evaluated, with the purchasability parameter defined as the most critical indicator of freshness. Using seven popular transfer learning algorithms (EfficientNetB0, ResNet50, DenseNet121, VGG16, InceptionV3, MobileNet, and VGG19), 2464 predictive models (1120 classification and 1344 regression) were trained. Classification models were evaluated using accuracy, precision, recall, F1-score, and response time, while regression models were assessed using mean absolute error and tolerance-based error metrics. The results showed that the MobileNet algorithm achieved the best overall performance, successfully predicting 15 of the 22 parameters with the lowest error or highest accuracy. Importantly, in the prediction of the most critical parameter—purchasability—the DenseNet121 architecture yielded the best classification performance with an accuracy of 0.9894. The findings indicate that deep learning-based image analysis is a viable method for evaluating the freshness of fish.

## 1. Introduction

Fish and seafood are nutrient-rich foods, abundant in high-quality protein, omega-3 fatty acids, and various vitamins and minerals. However, due to their structural characteristics, they are highly prone to spoilage. Traditional freshness assessment methods such as sensory evaluation, microbiological counts, and chemical tests (e.g., TVB-N, TBA, K-value) can be time-consuming, destructive, and subjective, and require complex laboratory equipment [1]. In recent years, artificial intelligence (AI) and computer vision technologies have emerged as promising tools for non-invasive, rapid, and objective quality assessment in food products. Image-based methods have attracted significant interest due to their non-destructive nature, potential low cost, and high accuracy. These methods typically focus on specific parts of the whole fish (eyes, gills, skin) and use deep learning architectures such as Convolutional Neural Networks (CNNs) for classification. Particularly in seafood processing, image-based models offer a unique advantage by enabling real-time monitoring of external quality attributes such as skin brightness, texture, and discoloration. Several studies have demonstrated the feasibility of using convolutional neural networks (CNNs) to predict specific freshness indicators in fish and meat products. However, the existing literature generally remains limited to single-angle (top view) and single-parameter classification frameworks, often using constrained imaging conditions. Consequently, a clear research gap persists in developing models capable of integrating multi-angle visual information together with multiple physicochemical and sensory indicators.

This study directly addresses this gap by implementing a multi-angle, multi-parameter prediction framework that enables a more comprehensive and robust evaluation of fish freshness compared to conventional single-angle or single-feature approaches.

The eyes and gills of fish are considered the most prominent indicators of freshness. Yildiz et al. [2] developed a mobile application that uses SqueezeNet and VGG19 models as feature extractors and ML classifiers such as K-NN, RF, SVM, LR, and ANN to analyze fish eye images. Issac et al. [3] proposed an automatic method for segmenting fish gills using active contour and thresholding techniques, and a freshness assessment model based on statistical features in the saturation channel. Larger and more complex datasets have unlocked the power of deep learning. Yasin et al. [4] used SqueezeNet and InceptionV3 on a dataset of 4476 fish images and reported that SVM, ANN, and LR models achieved 100% accuracy for each deep learning method. Taheri-Garavand et al. [5] used a VGG-16-based CNN model to classify common carp images, achieving 98.21% accuracy. Choudhury et al. [6] evaluated four pre-trained CNN models (VGG, ResNet, Inception and MobileNet) for grading the freshness of Mourala fish and reported acceptable results. AI is also widely used for fish species recognition. Rahman et al. [7] used seven CNN architectures (DenseNet121, EfficientNetB0, ResNet50, VGG16, VGG19) and seven ML classifiers to classify thirteen fish species, achieving up to 100% accuracy for both binary (freshwater/saltwater) and multiclass classification. Ergün [8] proposed the SwinFishNet model, based on Swin Transformer, for fish species classification, achieving accuracies between 98.47% and 99.64% on three different datasets. Banerjee et al. [9] used autoencoder-based deep learning models to classify major Indian carp species with 97.33% accuracy.

Setyagraha et al. [10] created a comprehensive dataset (ESF) comprising 336,997 individual sensor measurements—each representing a one-second e-nose reading collected during the spoilage process of five major seafood species (tuna, salmon, cod, shrimp, and crab). Ref. [11] evaluated seven ML algorithms with hyperparameter optimization and reported that the K-NN algorithm showed exceptional performance for classifying seafood freshness and predicting microbial populations. Grassi et al. [12] used a portable e-nose consisting of four metal oxide semiconductor sensors, a photoionization detector, and two electrochemical cells to measure sole, red mullet, and cuttlefish throughout their shelf life, demonstrating that the K-NN model achieved 100% overall sensitivity, specificity, and precision. Kumaravel et al. [13] used a Random Forest (RF)-based prediction model to evaluate the effectiveness of vacuum, shrink, and normal packaging. They reported the lowest MSE values for Pomfret and Mackerel (0.004625 and 0.005034, respectively). Li et al. [14] quantitatively assessed the freshness of frozen horse mackerel using ANN, Extreme Gradient Boosting (XGBoost), Random Forest Regression (RFR), and Support Vector Regression (SVR).

Qin et al. [15] used multimodal hyperspectral imaging techniques (VNIR reflectance, UV-induced fluorescence, SWIR reflectance, Raman) to detect fish fillet substitution and mislabeling. Hardy et al. [16] used hyperspectral imaging and optimized K-NN analysis to classify the freshness state of salmon fillets, achieving an average classification accuracy of 77.0%. Ortega et al. [17] used HSI to determine the shelf life of Atlantic cod (*Gadus morhua* L.) with high efficiency. Kashani Zadeh et al. [18] worked on farmed and wild salmon using multimodal spectroscopy (VIS-NIR, SWIR reflectance, and fluorescence) and data fusion. Dudnyk et al. [19] developed an edible sensor from pectin and red cabbage (materials derived solely from food) and observed clear color changes with spoilage in beef, chicken, shrimp, and fish samples. Wang et al. [20], in collaboration with the Sydney Fish Market (SFM), developed a blockchain-enabled system (BeFAQT) for fish provenance and quality tracking. The system utilized IoT and AI technologies including NB-IoT, image processing, and biosensing to provide real-time objective fish quality assessment. Ismail et al. [21] proposed a blockchain-based fish supply chain framework to maintain fish quality and authenticity.

Chiang [22] designed an IoT-based fish meat freshness detector for seafood market applications. Sengar et al. [23] proposed a non-destructive framework for fish freshness identification based on fish skin tissue, extracting statistical features in the HSV color space and achieving a maximum classification accuracy of 96.66%. The current literature clearly demonstrates that Artificial Intelligence and Machine Learning have revolutionized the assessment of fish freshness. Various technologies such as image processing, hyperspectral imaging, and smart sensors offer superior accuracy, speed, and objectivity compared to traditional methods. Integration with IoT and blockchain further enhances transparency and traceability across the supply chain.

This study addresses a key gap in automated seafood quality assessment by developing a deep learning framework capable of predicting 22 sensory and colorimetric quality parameters of sea bass (*Dicentrarchus labrax*) from multi-angle images captured with and without background. The primary aim is to evaluate whether image-based models can reliably predict these parameters—particularly purchasability, the industry’s operational decision criterion. To this end, 2464 models were trained using seven transfer learning architectures and assessed in terms of accuracy, mean absolute error, and inference time. The study tests three hypotheses: (1) deep learning models can predict both categorical and numerical quality attributes with high fidelity; (2) model performance varies significantly across transfer learning architectures; and (3) background presence influences predictive accuracy.

## 2. Materials and Methods

### 2.1. Materials

The material used in this study consisted of images of sea bass (*Dicentrarchus labrax*). The fresh fish were obtained from a local fish market and immediately transported to the laboratory in ice. The fish had died before being used. The fish samples were analyzed over a 21-day storage period. During this time, the samples were stored on ice to maintain freshness and simulate typical cold storage conditions. All measurements were conducted in a laboratory environment with a controlled room temperature of 20 ± 2 °C. Daily quality analysis and image acquisition were conducted for each fish, and the resulting quality data and images collectively formed the dataset for this study. The quality analysis was performed in two categories: color measurement by using a CM-400 device and raw sensory evaluation by the Quality Index Method proposed by Bonilla et al. [24] with minor modification. The parameters and their respective evaluation criteria recommended for fish products are presented in Table 1. The last two parameters in the table are computed based on the values of the other parameters. If the total score of the parameters is below 7, the fish is considered unspoiled and labeled as acceptable for purchase. Conversely, if the total score exceeds 7, the fish is considered spoiled and labeled as unsuitable for purchase.

The sensory evaluation was conducted by a panel of ten trained panelists who were familiar with the sensory attributes of the samples. Panelists consisted of ten volunteer females and males (aged from 25 to 55). The panel verbally consented to take part in this research. To ensure panelist consistency, all participants underwent a training session prior to the evaluation, during which they were introduced to the scoring criteria adapted from Bonilla et al. [24]. The samples were handled under hygienic conditions, and no tasting or ingestion of the fish was involved; only visual and olfactory evaluations were conducted. Therefore, the study did not pose any safety risks to the participants. In addition, inter-rater reliability was assessed using Cronbach’s alpha, which yielded a value of 0.87, indicating a high level of agreement among panelists.

Color analysis was conducted using the method described by Calder [25]. In this analysis, the L*, a*, and b* values-representing the three coordinates of the CIELab color system-were recorded using a CR-400 device (Konica Minolta, Osaka, Japan). The ‘L*’ value indicates lightness (ranging from 0 for black to 100 for white); the ‘+a*’ value represents red, while ‘−a*’ indicates green (within a range of −60 to +60); similarly, the ‘+b*’ value denotes yellow and ‘−b*’ represents blue (also within a range of −60 to +60).

Based on the measured L*, a*, and b* values in the CIELab system, the whiteness, chroma, and hue values were calculated using the formulas provided below (Equations (1)–(3)) as described by Hunter [25].

(1) Whiteness (W) = 100 − √ ((100 − L*)^2^ + a*^2^ + b*^2^)

(2) Chroma (C*) = √ (a^2^ + b^2^)

(3) Hue angle (h°) = arctan(b*/a*)

All measurements were taken from three different points on the fish—dorsal, terminal, and ventral—to represent the color scale. Since the measured L*, a*, and b* values, as well as the calculated whiteness, chroma, and hue values, are parameters that indicate the freshness of the fish, estimating the average of these values also constitutes one of the objectives of this study.

### 2.2. Imaging System and Lighting Conditions

To minimize shadow formation on the samples, illumination was provided from three lines: one positioned at a 90° angle (perpendicular to the base) and two positioned at 45° angles (dorsal and ventral). To ensure consistency in illumination, 5050 and 2835 SMD LEDs with a color temperature of 3000 K were used, with a density of 3 LEDs per 5 cm. The experimental room was isolated from daylight to maintain constant lighting conditions.

**Figure 1:**
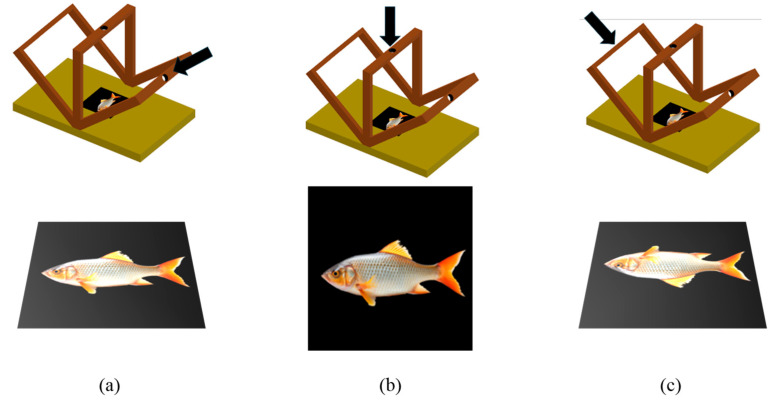
Imaging setup used for photographing fish samples: (**a**) right angle view; (**b**) top view; (**c**) left angle view.

The setup consisted of wooden stands (10 cm width) with circular apertures in the center to accommodate the camera lens (Figure 1). LED strips were fixed along the length of the stands, circling these apertures. To prevent any angular deviation over time, the stands were secured at multiple points. The distance from the camera lens to the sample was fixed at 45 cm for all angles.

Images were captured using a Canon EOS 60D camera (Canon Inc., Tokyo, Japan) equipped with an APS-C CMOS sensor and an 18–200 mm VR lens kit. The lens lock was engaged at the “0” zoom position to ensure identical magnification in all photographs. Based on the background color and light intensity, the shooting parameters were set to an Aperture of F/4.5, Shutter Speed of 1/50, and ISO 100. The captured images had a resolution of 3456 × 2308 pixels. Data collection was performed once daily (morning hours) by capturing a single shot for each angle.

Following the daily quality analysis, the imaging process was carried out. A specialized photography setup was prepared for this purpose. Within this setup, each fish was photographed from three different angles. The imaging environment is shown in Figure 1.

**Figure 2:**
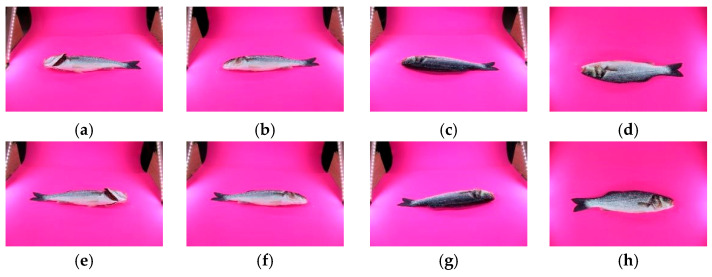
Example image dataset of a single fish captured from multiple viewpoints: (**a**) left-side angled view with gills open, (**b**) left-side angled view with gills closed, (c) left-side angled rear view, (**d**) top view of the left side, (**e**) right-side angled view with gills open, (**f**) right-side angled view with gills closed, (**g**) right-side angled rear view, and (**h**) top view of the right side of the fish.

In this imaging setup, both sides of each fish were photographed from three different angles. Additionally, side-perspective images were captured with the gills clearly visible. As a result, a total of eight distinct images were obtained for each fish. An example image dataset for a single fish is presented in Figure 2.

For each fish sample, quality analysis and image acquisition were conducted daily for a period of 21 days, primarily during the morning hours. During the later stages of spoilage, images were additionally taken at noon and in the evening. In the first 8 days, only one image per angle was captured each day, while from Day 9 onward three images per angle were recorded to better document the progression of deterioration.

**Figure 3:**
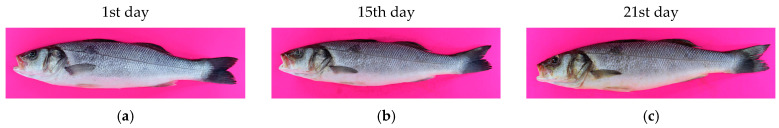
Representative images of the same fish captured on three different storage days—(**a**) day 1, (**b**) day 15, and (**c**) day 21—illustrating the visual progression of spoilage.

Figure 3 presents representative images of the same fish captured at three different storage days, ranging from fresh to visibly spoiled. This temporal sequence allows a direct visual comparison of freshness degradation, revealing gradual changes in color, glossiness, and surface characteristics.

A total of 10 fish were used in the study, and for each imaging angle, 470 images were collected along with the corresponding quality analysis data at the time of capture. Thus, each imaging angle was treated as a separate dataset, resulting in a total of eight distinct datasets.

To enhance the image quality and reduce background interference, a pink background-significantly different from the color characteristics of the fish-was initially used during the imaging process. However, to further isolate the fish features, the backgrounds in all images were removed, and background-free versions of the datasets were created. Consequently, the study produced 8 datasets with backgrounds and 8 datasets without backgrounds, totaling 16 datasets.

For background removal, the “rembg” library which is an open-source Python 3.11.4 package, was utilized. This library employs a machine learning model trained on a large image dataset to accurately separate the foreground object from the background. Rembg is built on U^2^-Net, a deep learning architecture specifically designed for salient object detection. By analyzing image pixels and classifying them as foreground or background, it leverages the power of convolutional neural networks. As a result, it enables fast and accurate background removal, making it highly suitable for applications such as product photography, image preprocessing, and visual data analysis [26,27]. The rembg, developed by Gatis [28], was employed for the mentioned purpose.

Each dataset name follows the structure XXX_WWW_YY_ZZ, where each component describes a specific imaging condition. The first part, XXX, indicates whether the background is present (WBG = with background) or removed (BGF = background-free). The second part, WWW, specifies the viewing type, indicating whether the image is taken from an angle (ANG) or from above (UPS). The third component, YY, denotes the side of the fish being photographed (right (RT) or left (LF)). The final part, ZZ, identifies the exact angle or gill condition: values such as 01 represent the front view with gills open, 02 indicate the front view with gills closed, and BK refers to the dorsal (back) angle. For the top viewed data sets the ZZ part is always 01 and it does not point any angle or issue about gill condition. This coding structure consistently labels all eight image types used in the study and is summarized in Table 2. All coded datasets are visualized and summarized in Appendix A.

### 2.3. Deep Learning Models

In this study, artificial intelligence applications were employed to predict the quality parameters of a fish based on its image, with particular emphasis on the most critical parameter which is purchasability. The quality parameters were categorized into two groups: categorical (discrete-valued) parameters (sensory analysis) and numeric (continuous-valued) parameters (color analysis). A classification model was developed for the prediction of categorical parameters, while a regression model was established for the prediction of numeric parameters. Although it is also possible to convert continuous freshness indicators into discrete classes (e.g., “low,” “medium,” “high”), such binning procedures may lead to information loss by masking subtle but meaningful variations in colorimetric measurements. Because the color parameters used in this study (L*, a*, b*, whiteness, chroma, hue) are continuous and highly sensitive to early-stage deterioration, preserving their numeric structure enables more precise modeling of freshness-related changes. Therefore, regression was preferred over discretization to maintain measurement resolution and provide more accurate predictions of continuous quality parameters. For both prediction tasks, seven different transfer learning models were utilized: EfficientNetB0, ResNet50, DenseNet121, VGG16, InceptionV3, MobileNet, and VGG19. The use of these transfer learning models was motivated by existing research demonstrating their strong performance in image processing applications. Architectures such as DenseNet and ResNet enable deep feature propagation and extraction of micro-level texture cues, while EfficientNet and MobileNet offer optimized feature scaling that enhances sensitivity to gradient-based color changes. Similarly, VGG and Inception structures provide strong hierarchical representations that effectively capture brightness, discoloration, and surface texture—attributes that are critical when assessing fish quality from images. These characteristics collectively make the selected architectures well aligned with the visual challenges inherent in seafood freshness prediction.

EfficientNet-B0, introduced by Tan and Le [29], represents the baseline model of the EfficientNet architecture. Unlike traditional networks such as ResNet or VGG, the EfficientNet family is designed to achieve state-of-the-art accuracy while using significantly fewer parameters and computational operations (FLOPs). EfficientNet-B0 in particular emphasizes a balanced compromise between model complexity and predictive performance. Its architectural design begins with an input layer that processes images of size 224 × 224 pixels with three RGB channels, a commonly adopted resolution in computer vision tasks. This is followed by a series of convolutional layers utilizing 3 × 3 kernels in the initial stage, alongside batch normalization and the Swish (SiLU) activation function [29,30].

ResNet-50 is a deep convolutional neural network architecture consisting of 50 layers organized around a bottleneck residual design introduced by He et al. [31]. This model adopts a sequence of convolutional, pooling, and fully connected layers, yet its defining architectural feature is the use of residual blocks with identity or projection shortcut connections. Each residual block contains a three-layer bottleneck structure—comprising a 1 × 1 convolution for dimensionality reduction, a 3 × 3 convolution for spatial feature extraction, and another 1 × 1 convolution for restoring channel depth—which significantly reduces computational cost while enabling deeper model construction. ResNet-50 follows a hierarchical arrangement of these bottleneck blocks across four stages, progressively increasing the number of channels while decreasing the spatial resolution. This architectural design enables the network to learn highly discriminative features efficiently, achieving strong performance across a wide range of image recognition tasks [31,32].

DenseNet-121, proposed by Huang et al. [33], follows the principle of Densely Connected Convolutional Networks (DenseNets), which introduce a connectivity pattern to address the vanishing gradient problem while maximizing information and gradient flow across the network. In this architecture, each layer receives as input the concatenated feature maps of all preceding layers and contributes its own output to all subsequent layers. This design promotes feature reuse and reduces redundancy, thereby lowering the overall parameter count compared to other deep networks. DenseNet-121 consists of alternating dense blocks and transition blocks: within each dense block, the spatial dimensions of feature maps remain constant to allow concatenation, while their depth increases; transition blocks, by contrast, downsample the feature maps using 1 × 1 convolutions and 2 × 2 pooling. A key hyperparameter of the model is the growth rate, which determines how many new feature maps each layer contributes, effectively controlling the information flow throughout the network. By combining depth with efficient feature utilization, DenseNet-121 achieves state-of-the-art performance on object recognition benchmarks, with fewer parameters and lower computational costs than many comparable architectures [33].

The VGG-16 convolutional neural network (CNN) architecture, introduced by Simonyan and Zisserman [34], achieved a test accuracy of 92.77% on the ImageNet benchmark, which contains approximately 14 million images across 1000 categories. It accepts input images of size 224 × 224 × 3 and employs a deep sequence of convolutional and max-pooling layers, beginning with two convolutional layers followed by a pooling layer, then two more convolutional layers and another pooling operation, followed by three convolutional layers and a pooling layer, repeated twice, and concluding with a final block of three convolutional layers and a pooling layer. Each convolutional layer uses 3 × 3 filters with a stride of 1, while max-pooling layers use 2 × 2 filters with a stride of 2. After the final pooling stage, the network includes three fully connected layers with rectified linear unit (ReLU) activation, culminating in a softmax classifier for multi-class prediction. In total, the model consists of 16 weight layers (13 convolutional and 3 fully connected), and its relatively small 3 × 3 receptive field enables the capture of fine-grained spatial features while supporting deep representational learning [34,35,36].

The InceptionV3 model was developed by Szegedy et al. [37] at Google Research as an extension of the original GoogLeNet architecture. Structurally, it is a 42-layer deep convolutional neural network that introduces several innovations to improve both accuracy and efficiency. Instead of simply stacking more layers, InceptionV3 factorizes larger convolutions (such as 7 × 7) into smaller 3 × 3 convolutions, and applies grid reduction techniques to progressively downsample feature maps—from 35 × 35 with 288 filters, to 17 × 17 with 768 filters, and finally to 8 × 8 with 1280 filters—before producing a final concatenated output of 2048 filters. The model incorporates auxiliary classifiers to stabilize training, extensive use of batch normalization, and optimized inception modules, which together allow it to achieve high performance. Despite being deeper than its predecessors, InceptionV3 is only about 2.5 times more computationally expensive than GoogLeNet, while remaining significantly more efficient than architectures like VGGNet [37,38].

MobileNet is a lightweight CNN architecture optimized for mobile and embedded applications. Its key structural innovation is the use of depthwise separable convolutions, where each input channel is filtered independently through a depthwise convolution and then combined using a 1 × 1 pointwise convolution. This design reduces computational cost by up to 8–9 times compared with standard convolutions while maintaining competitive accuracy. MobileNet also includes two hyperparameters—the width multiplier and resolution multiplier—which allow the network to scale in size and computational load. Each convolutional stage is followed by batch normalization and ReLU activation, with downsampling achieved through strided layers. The architecture ends with global average pooling and a softmax classifier, forming a compact 28-layer model suitable for resource-constrained environments [39].

VGG19, proposed by the Visual Geometry Group (VGG) at the University of Oxford in 2014, is a deep learning model that gained prominence through its remarkable performance in the ImageNet competition. As a deeper version of VGG16, this architecture consists of 19 layers in total 16 convolutional and 3 fully connected and employs sequential 3 × 3 convolution filters combined with max-pooling operations to effectively learn complex visual features. Its deep structure enhances generalization capacity, making it a powerful and widely adopted model in both research and industrial applications for tasks such as image classification and object detection [40,41,42].

### 2.4. Modeling

As summarized in Table 3, the study utilized images from 16 different datasets as inputs, employed 7 distinct prediction algorithms for model training, and generated outputs for 22 quality parameters. In total, 1120 classification models (16 datasets × 7 models × 10 categorical parameters) and 1344 regression models (16 datasets × 7 models × 12 numerical parameters) were developed.

Among the output parameters used in the models, Avg_V_L represents the average L* value measured vertically from three points, while Avg_D_L corresponds to the horizontal average. The same approach applies to other color parameters.

The essential training configurations applied across all regression and classification models are summarized in Table 4. Since each of the seven transfer-learning architectures was trained under the same experimental settings, the table provides a clear and concise overview of the data split, optimization setup, loss functions, evaluation metrics, and overfitting prevention measures used in the study.

To evaluate the classification models, four performance metrics were used: Accuracy, Precision, Recall, and F1-Score. The formulas for these metrics are presented in Equations (4)–(7), where TP denotes true positives, TN true negatives, FP false positives, and FN false negatives [43].

(4) $$Accuracy=\frac{TP+TN}{TP+TN+FP+FN}$$

(5) $$Precision=\frac{TP}{TP+FP}$$

(6) $$Recall=\frac{TP}{TP+FN}$$

(7) $$F1-Score=2\times \frac{Precision\times Recall}{Precision+Recall}$$

In the regression models, the predicted parameters L*, a*, and b* are highly sensitive. Therefore, for models developed to predict these parameters, performance was evaluated using tolerance-based error metrics. These metrics assess prediction accuracy by categorizing the absolute differences between predicted and actual values into predefined tolerance ranges. The tolerance intervals and corresponding error classes adapted form [44] for L*, a*, and b* are defined as follows:

• The prediction is Excellent if the absolute error
  ○ is ≤ 0.1 for L*, a*, and b*.
• The prediction is Good if the absolute error
  ○ is between 0.1 and 0.5 for L*,
  ○ is between 0.1 and 0.3 for a* and b*.
• The prediction is Medium if the absolute error
  ○ is between 0.5 and 1.0 for L*,
  ○ is between 0.3 and 0.6 for a* and b*.
• The prediction is Acceptable if the absolute error
  ○ is within 0–1.0 for L*,
  ○ is within 0–0.6 for a* and b*.
• The prediction is Reject if the absolute error
  ○ exceeds 1.0 for L*,
  ○ exceeds 0.6 for a* and b*.

In addition to the tolerance-based metrics, Mean Absolute Error (MAE) was also employed to evaluate the performance of all regression models, including those predicting L*, a*, and b* values. MAE measures the average magnitude of the errors between predicted and actual values, regardless of their direction. It provides a straightforward and interpretable indication of prediction accuracy, with lower values indicating better performance. The formula used to calculate MAE is given in Equation (8), where $y_{i}$ represents the actual value, ${\hat{y}}_{i}$ the predicted value, and n the number of observations [45].

(8) $$MAE=\frac{1}{n}\sum_{i=1}^{n}\left|y_{i}-{\hat{y}}_{i}\right|$$

For both categories of models, the prediction time (response time) in seconds was also calculated as an additional performance metric. All models were executed using Python 3.11.4, and the findings and results are presented in detail in the following sections.

## 3. Results and Discussion

Model performances were evaluated across multiple datasets and using various performance metrics, including Accuracy, Precision, Recall, F1-Score, tolerance-based error rates, and Mean Absolute Error (MAE). Results are reported separately for classification and regression tasks to provide a clearer comparison of model effectiveness.

The results obtained from the classification predictions were analyzed, and for each parameter, the top 10 models with the highest accuracy scores are listed in Appendix B. When the classification results were aggregated and analyzed in terms of parameter-based predictability, Skin Mucus and Scale emerged as the most accurately predicted parameters, both achieving a perfect accuracy score of 1.000. These were followed by Purchasability, which also demonstrated high predictive performance (Accuracy = 0.989), indicating its visual consistency and its suitability for image-based assessment. On the other hand, Total Score, Skin Odor, and Gill Odor showed the lowest accuracy levels, suggesting greater complexity or less visually distinctive cues associated with these parameters.

In terms of algorithmic performance, DenseNet121 and MobileNet each accounted for the highest accuracy in four parameters, while EfficientNetB0 was the top-performing model in two cases—both of which yielded perfect scores. These findings confirm the strong generalization capabilities of DenseNet121 and the computational efficiency of MobileNet, while also highlighting the specialized effectiveness of EfficientNetB0 in classifying highly distinguishable features. A summary of parameter-level accuracies and the corresponding best-performing algorithms is presented in Table 5.

An analysis of the top-performing datasets for each classification parameter (Table 6) reveals that a limited number of datasets consistently yielded the highest prediction accuracy across multiple quality indicators. Specifically, the datasets coded as BGF_ANG_LF_01 and WBG_ANG_RT_BK each ranked first in three different parameters, indicating their high-quality imaging angles and visual clarity. Notably, BGF_ANG_LF_01 coded dataset was the best-performing dataset for Scale, Purchasability, and Total Score, while WBG_ANG_RT_BK coded dataset produced the highest accuracy for Purchasability, Skin Texture, and Gill Mucus.

The dataset with code WBG_ANG_RT_02 followed closely, providing the best results for Skin Glossiness and Gill Color, both of which are highly dependent on light reflection and color balance-factors likely well-captured by this angle. In contrast, BGF_ANG_RT_01 and WBG_ANG_LF_02 coded datasets, each appeared as the top dataset for only one parameter (Gill Odor and Skin Odor, respectively).

These results imply that dataset quality, particularly in terms of angle and background conditions, plays a critical role in enhancing the performance of classification models across different freshness indicators. As indicated by the findings presented in Table 5 and Table 6, the most effective models for the Purchasability, Skin Texture, Skin Brightness, Gill Colour, Gill Mucus, and Total Score parameters were developed using with background images. An examination of the model prediction results for the ten best models presented in Appendix B reveals that background images yielded superior results in seven of the ten models that produced the most accurate predictions for Skin Glossiness, Gill Mucus, Skin Texture, and Gill Odour. In a similar vein, predictions regarding the Skin Mucus and Scale Arrangement metric yielded favorable outcomes in five of the ten models. Likewise, the Skin Odor metric demonstrated successful predictions in eight of the ten models. Furthermore, predictions concerning the Gill Colour and Total Score metrics yielded positive results in six of the ten models. Finally, predictions regarding the Purchasability metric yielded successful results in nine of the ten models, once again underscoring the efficacy of background images in facilitating accurate predictions. This finding suggests that while the background does have a positive effect on the models’ results, it does not have a significant impact on the best model. An evaluation of the image angles reveals that the RT_BK angle, which corresponds to the location of the dorsal scales, demonstrates efficacy in relation to Purchasability, Skin Texture, and Gill Mucus. In contrast, the LF_01 and 02 angles exhibit effectiveness in relation to other parameters.

The results obtained from the prediction of the L*, a*, and b* parameters were analyzed, and for each parameter, the top 10 models with the lowest Mean Absolute Error (MAE) values are presented in Table 7.

The results obtained from the prediction of Whiteness, Chroma, and Hue parameters were analyzed, and for each parameter, the top 10 models with the lowest Mean Absolute Error (MAE) values are listed in Table 8.

According to the findings presented in Table 7, the regression model can be utilized to make predictions with a minimal mean absolute error (MAE) for Avg V_b, Avg D_b, Avg V_a, and Avg D_a. Predictions can be made for Avg V_L and Avg D_L with relatively higher estimates. A similar outcome is evident in Table 8, where minimal MAE predictions are observed for Avg V_Croma, Avg V_Hue, Avg D_Croma, and Avg D_Hue, while comparatively elevated MAE predictions are noted for Avg V_Whiteness and Avg D_Whiteness. An analysis of the models demonstrating high prediction performance reveals that more successful predictions can be produced from background images. Upon evaluation of the image angles, it was ascertained that the RT_BK and LF_02 angles exert a favorable influence on the prediction performance. Consequently, an evaluation of image angles reveals that the dorsal scales and gills of fish have a discernible impact on coloration.

To contextualize the error values in terms of practical freshness assessment, it is important to note that chroma and hue values in fish typically change gradually during early preservation but exhibit more pronounced shifts as spoilage progresses. A Mean Absolute Error of approximately 3 units for chroma or hue corresponds to a small portion of the natural dynamic range observed in the colorimetric measurements collected in this study. Such differences generally reflect subtle visual variations that are unlikely to alter consumer-level judgments during the initial stages of storage. However, as fish approaches spoilage, chroma and hue shifts often exceed 10–15 units, meaning that a prediction error of ~3 units still preserves the model’s ability to reliably distinguish major freshness phases In this sense, an MAE near 3 remains within an acceptable practical tolerance and does not compromise the usefulness of the predictions for quality monitoring.

The aggregated analysis of all regression results revealed clear distinctions in parameter predictability. Among the twelve parameters evaluated, AVG_D_a, AVG_D_Chroma, and AVG_D_b showed the highest predictability with the lowest MAE values (0.205, 0.443, and 0.694 respectively), indicating strong model performance in estimating horizontal color components. Conversely, parameters such as AVG_V_Whiteness, AVG_D_Whiteness, and AVG_D_L had the highest MAE values, suggesting greater variability or complexity in modeling vertical whiteness and lightness characteristics.

In terms of model performance, MobileNet was identified as the most effective regression algorithm, achieving the lowest MAE for 11 out of 12 parameters. This highlights MobileNet’s strong generalization capability and suitability for real-time predictive tasks. VGG16, in contrast, ranked first for only one parameter (AVG_V_L), indicating more limited but potentially specialized performance. These results are summarized in Table 9.

An evaluation of the datasets used in the best-performing regression models highlights the impact of image perspective on model accuracy. As shown in Table 10, the dataset with code WBG_ANG_RT_BK yielded the best results for four different parameters-including AVG_D_b, AVG_V_b, AVG_V_L, and AVG_V_Whiteness-indicating its strong suitability for predicting both chromatic and background-related values. Similarly, dataset with code BGF_ANG_LF_02 ranked first for three parameters, particularly excelling in the prediction of lightness-related components such as AVG_D_L and AVG_D_Whiteness.

In contrast, datasets captured from top-down views (e.g., WBG_UPS_LF_01) appeared only once, reinforcing the observation that side-angle images provided richer visual information for regression-based estimation tasks. Overall, lateral imaging angles-especially those from the right and left sides-were more frequently associated with higher prediction accuracy, suggesting their effectiveness in revealing surface-level and structural features critical for quality estimation.

When classification and regression models were evaluated together across all 22 parameters, MobileNet emerged as the most effective algorithm, providing the best predictions for 15 parameters. This was followed by DenseNet121, which performed best for 4 parameters, while EfficientNetB0 and VGG16 each ranked first for 1 parameter. These results demonstrate that MobileNet not only generalizes well across classification and regression tasks but also offers computational advantages with low response times, making it ideal for real-time quality assessment scenarios.

From the dataset perspective, the best overall performance was consistently achieved by the dataset coded WBG_ANG_RT_BK, which contains side-angle images with background, specifically showing the dorsal (back) side of the fish. This suggests that the visibility of surface textures and contours from this perspective provided the most informative visual cues for both classification and regression models.

According to the findings, the freshness of the fish, the results of the sensory analysis, and the color and texture were predicted with a high degree of success. Examining different CNN models revealed that artificial neural network models using MobileNet and VGG16 algorithms produced the best results. Various studies in the literature have examined classifying fish as fresh or spoiled. Similarly, in the study by Yildiz et al. (2024) [2], models effectively captured visual indicators of freshness deterioration using image data focused on the eye region. Of the configurations tested, the combination of VGG19 for feature extraction and an artificial neural network (ANN) for classification achieved the highest accuracy of 77.3% on the FFE dataset. These results demonstrate that VGG19’s deep features contain highly discriminative information related to fish freshness that can be efficiently interpreted by ANN models. The results show that hybrid models outperform traditional single-method approaches in visual quality assessment tasks. Similarly, the current study shows that integrating deep learning and ANN algorithms yields good results.

The literature has examined predictions made using CNN models with different methods. While artificial neural networks have generally produced the best results, the SVM method has also yielded good results in some cases. Yasin et al. (2023) [4] classified 4476 fish as fresh or stale using SqueezeNet and InceptionV3 algorithms. They achieved 100% accuracy in predictions using the Support Vector Machine (SVM) algorithm. Isaac et al. (2017) [3] made predictions by segmenting pixels belonging to the gill region in the image from the “a” channel of an image converted to LAB space. They examined the effect according to days, similar to this study.

The datasets were intentionally kept separate because one objective of the study was to evaluate the predictive contribution of each imaging angle independently. Different angles highlight different freshness-related cues—such as dorsal texture, mucus patterns, or color gradients—and combining all images into a single dataset would obscure these angle-specific effects. This separation allowed clearer interpretation of which perspectives were most informative. As future work, unified models trained on the full dataset will be explored to complement the angle-specific results reported here.

The superior performance of the proposed system—particularly in multi-parameter predictions—can be attributed to several characteristics of the imaging protocol and dataset design. First, the dorsal-region images (e.g., WBG_ANG_RT_BK), which consistently ranked among the best-performing datasets, provide enhanced visibility of surface texture, mucus accumulation, and color gradients along the lateral line. These features are directly associated with both sensory parameters (e.g., skin texture, gill mucus) and continuous colorimetric values, enabling the models to extract richer and more discriminative visual cues. Second, the multi-angle acquisition ensured that subtle freshness-related changes—such as progressive dullness, gill discoloration, or softening of the skin—were captured from perspectives that maximize contrast and structural detail. Compared to earlier studies that relied predominantly on eye or gill regions, the inclusion of full-body views, especially from dorsal and side angles, exposes a more comprehensive set of deterioration indicators. This broader visual context allows the transfer learning models to generalize across multiple parameters simultaneously, explaining why the system achieved stronger predictive performance than approaches focused on single-region or single-parameter assessment.

## 4. Conclusions

This study demonstrated the potential of deep learning-based image analysis in predicting the freshness of sea bass through visual quality indicators. A total of 22 quality parameters—10 categorical and 12 numerical—were defined to represent fish freshness, with particular emphasis on the purchasability parameter as the most critical outcome. Using a structured dataset comprising 16 image variations (captured from 8 angles, with and without background), a total of 2464 predictive models were developed using seven state-of-the-art transfer learning architectures.

The primary limitations of this study are summarized below. In this study, time-dependent data were collected for 10 fish of the same species, and images were obtained accordingly. Sensory and colorimetric data were obtained through expert opinions and spectrophotometric analysis.

The findings revealed that side-view images, especially those showing the dorsal side, provided the most informative visual cues. Among the models, DenseNet121 achieved the highest performance in predicting purchasability (accuracy = 0.9894), while MobileNet demonstrated superior generalization across multiple parameters, being the best-performing algorithm for 15 out of 22 features.

On the other hand, some parameters such as Total Score, Skin Odor, and Gill Odor exhibited lower classification accuracy, likely due to their subjective nature or limited visual distinguishability. These findings suggest that certain freshness attributes may require multimodal approaches beyond image-based analysis for more accurate prediction.

Additionally, the comparison between datasets revealed that background-free and top-down images performed less effectively, whereas lateral images with a visible surface structure significantly enhanced prediction accuracy. This emphasizes the importance of imaging protocol and dataset design in AI-based food quality monitoring.

In conclusion, the study confirms that visual-based artificial intelligence systems can reliably estimate key freshness parameters in fish. These results point toward promising applications in automated seafood quality assessment and intelligent supply chain management. Future research may explore larger sample sizes, real-time monitoring, or the integration of other sensory modalities (e.g., odor or temperature sensors) to improve predictive accuracy in more complex or ambiguous cases.

## Figures and Tables

**Table 1:** Quality parameters and their evaluation criteria adapted from Bonilla et al. [24].

Quality Parameter	Score	Description
Skin Brightness	0	Iridescent
	1	Slightly dull
	2	Dull
Skin Mucus	0	Low and transparent
	1	Excessive and yellowish
Scale	0	Regular
	1	Disordered
Skin Texture	0	Firm
	1	Slightly soft
	2	Very soft
Skin Odor	0	Fresh and neutral
	1	Algae-like
	2	Sour milk
	3	Acetic and ammonia-like
Gill Color	0	Characteristic (bright red)
	1	Slightly brown
	2	Dark brown
Gill Mucus	0	No mucus
	1	Slight mucus
	2	Heavy mucus
Gill Odor	0	Fresh and neutral
	1	Algae-like
	2	Sour milk
	3	Acetic and ammonia-like
Total Score	0–7	Not Spoiled
	7–15	Spoiled
Purchasability	1	Yes
	2	No

**Table 2:** Description of dataset coding scheme used for fish images.

Code Component	Meaning	Values	Description
XXX	Background condition	WBG, BGF	WBG = with background BGF = background-free image
WWW	View type	ANG, UPS	ANG = angled view UPS = top (dorsal) view
YY	Fish side	RT, LF	RT = right side of the fish LF = left side of the fish
ZZ	Specific angle or gill condition	01, 02, BK	01 = front angle with gills open 02 = front angle with gills closed BK = back angle (dorsal direction)

**Table 3:** Summary of the model development framework used in the study.

Model Input		Model Algorithm		Model Output			
				Classification		Regression	
#	Dataset Code	#	Algorithm	#	Parameter	#	Parameter
1	BGF_ANG_LF_01	1	EfficientNetB0	1	Skin Brightness	1	Avg V_L
2	BGF_ANG_LF_02	2	ResNet50	2	Skin Mucus	2	Avg V_a
3	BGF_ANG_LF_BK	3	DenseNet121	3	Scale	3	Avg V_b
4	BGF_ANG_RT_01	4	VGG16	4	Skin Texture	4	Avg V_Croma
5	BGF_ANG_RT_02	5	InceptionV3	5	Skin Odor	5	Avg V_Hue
6	BGF_ANG_RT_BK	6	MobileNet	6	Gill Color	6	Avg V_Whiteness
7	BGF_UPS_LF_01	7	VGG19	7	Gill Mucus	7	Avg D_L
8	BGF_UPS_RT_01			8	Gill Odor	8	Avg D_a
9	WBG_ANG_LF_01			9	Total Score	9	Avg D_b
10	WBG_ANG_LF_02			10	Purchasability	10	Avg D_Croma
11	WBG_ANG_LF_BK					11	Avg D_Hue
12	WBG_ANG_RT_01					12	Avg D_Whiteness
13	WBG_ANG_RT_02						
14	WBG_ANG_RT_BK						
15	WBG_UPS_LF_01						
16	WBG_UPS_RT_01						

**Table 4:** Summary of Modeling Configuration for Regression and Classification.

Parameter	Regression	Classification
Models Used	EfficientNetB0, ResNet50, DenseNet121, InceptionV3, MobileNet, VGG16, VGG19	
Data Split	80% train/20% validation-test (test_size = 0.2, random_state = 42)	
Cross-Validation	Not applied	
Data Augmentation	None	
Hardware	CPU	
Loss Function	MSE	Categorical Cross-Entropy
Optimizer	Adam (default settings), batch size 8, 10 epochs	
Metrics	MAE	Accuracy, Precision, Recall, F1-Score
Overfitting Control	Frozen pretrained CNN backbone; only small dense head trained; short epoch count	

**Table 5:** Parameter-based classification accuracy and the best-performing algorithms.

Rank	Parameter	Accuracy	Algorithms		
			DenseNet121	MobileNet	EfficientNetB0
1	Skin Mucus	1.0000			✔
2	Scale	1.0000			✔
3	Purchasability	0.9894	✔		
4	Skin Texture	0.9043	✔		
5	Skin Brightness	0.8936		✔	
6	Gill Color	0.8936		✔	
7	Gill Mucus	0.8723	✔		
8	Gill Odor	0.8085		✔	
9	Skin Odor	0.7766	✔		
10	Total Score	0.5745		✔	
Count of being best model			4	4	2

**Table 6:** Distribution of best-performing datasets across quality parameters.

Parameter	Data Sets									
	BGF_ANG_LF_01	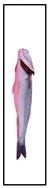	WBG_ANG_RT_BK	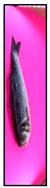	WBG_ANG_RT_02	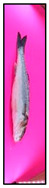	BGF_ANG_RT_01	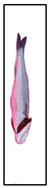	WBG_ANG_LF_02	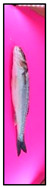
Skin Mucus	✔									
Scale	✔									
Purchasability			✔							
Skin Texture			✔							
Skin Brightness					✔					
Gill Color					✔					
Gill Mucus			✔							
Gill Odor							✔			
Skin Odor	✔									
Total Score									✔	
Count of being best	3		3		2		1		1	

**Table 7:** Top 10 regression models with the lowest MAE values for L*, a*, and b* predictions.

Parameter	Rank	Dataset	Algorithm	MAE	Excellent	Good	Medium	Reject	Accept	Response Time (s)
Avg V_L	1	WBG_UPS_LF_01	VGG16	2.849	2.128	11.702	12.766	73.404	26.596	0.576
	2	BGF_ANG_LF_BK	ResNet50	2.869	4.255	11.702	13.830	70.213	29.787	3.223
	3	BGF_UPS_LF_01	VGG16	2.877	2.128	8.511	9.574	79.787	20.213	0.801
	4	BGF_UPS_LF_01	VGG19	2.901	3.191	12.766	9.574	74.468	25.532	0.690
	5	BGF_UPS_RT_01	ResNet50	2.908	3.191	13.830	11.702	71.277	28.723	2.124
	6	WBG_ANG_RT_01	ResNet50	2.911	4.255	8.511	14.894	72.340	27.660	3.184
	7	BGF_ANG_RT_02	MobileNet	2.912	1.064	11.702	14.894	72.340	27.660	0.980
	8	BGF_ANG_RT_02	VGG19	2.921	6.383	11.702	17.021	64.894	35.106	0.608
	9	WBG_ANG_RT_BK	ResNet50	2.930	2.128	10.638	22.340	64.894	35.106	3.015
	10	WBG_ANG_LF_01	ResNet50	2.942	3.191	11.702	20.213	64.894	35.106	2.709
Avg V_a	1	WBG_ANG_RT_BK	MobileNet	0.805	11.702	57.447	22.340	8.511	91.489	1.164
	2	WBG_ANG_LF_BK	MobileNet	0.818	18.085	51.064	21.277	9.574	90.426	0.949
	3	WBG_ANG_LF_BK	InceptionV3	0.818	14.894	51.064	23.404	10.638	89.362	3.315
	4	WBG_ANG_LF_02	MobileNet	0.819	22.340	50.000	14.894	12.766	87.234	1.165
	5	WBG_ANG_RT_BK	DenseNet121	0.822	12.766	57.447	18.085	11.702	88.298	6.547
	6	BGF_ANG_RT_02	MobileNet	0.823	19.149	46.809	24.468	9.574	90.426	0.886
	7	WBG_ANG_RT_02	DenseNet121	0.826	17.021	51.064	22.340	9.574	90.426	6.103
	8	BGF_ANG_RT_02	DenseNet121	0.841	18.085	47.872	23.404	10.638	89.362	5.771
	9	BGF_UPS_LF_01	DenseNet121	0.846	13.830	52.128	22.340	11.702	88.298	4.943
	10	WBG_ANG_LF_02	DenseNet121	0.847	13.830	48.936	27.660	9.574	90.426	5.894
Avg V_b	1	WBG_ANG_LF_01	MobileNet	1.011	7.447	32.979	19.149	40.426	59.574	0.941
	2	WBG_ANG_LF_02	MobileNet	1.086	4.255	28.723	29.787	37.234	62.766	1.117
	3	WBG_ANG_RT_BK	MobileNet	1.113	7.447	25.532	23.404	43.617	56.383	1.126
	4	WBG_ANG_RT_01	MobileNet	1.157	7.447	28.723	21.277	42.553	57.447	0.977
	5	WBG_ANG_RT_02	MobileNet	1.172	8.511	20.213	23.404	47.872	52.128	0.902
	6	BGF_ANG_LF_02	InceptionV3	1.181	5.319	26.596	25.532	42.553	57.447	3.676
	7	WBG_ANG_LF_BK	MobileNet	1.202	9.574	22.340	19.149	48.936	51.064	0.949
	8	BGF_ANG_RT_02	InceptionV3	1.221	5.319	22.340	23.404	48.936	51.064	3.375
	9	WBG_ANG_LF_01	InceptionV3	1.235	3.191	27.660	21.277	47.872	52.128	3.591
	10	BGF_ANG_RT_01	DenseNet121	1.254	2.128	29.787	23.404	44.681	55.319	5.134
Avg D_L	1	BGF_ANG_LF_02	MobileNet	3.513	5.319	8.511	7.447	78.723	21.277	0.951
	2	BGF_UPS_RT_01	MobileNet	3.519	4.255	6.383	12.766	76.596	23.404	1.085
	3	BGF_ANG_RT_BK	MobileNet	3.551	2.128	9.574	13.830	74.468	25.532	0.885
	4	WBG_UPS_LF_01	MobileNet	3.569	1.064	6.383	10.638	81.915	18.085	1.142
	5	WBG_UPS_LF_01	DenseNet121	3.613	3.191	7.447	13.830	75.532	24.468	4.924
	6	BGF_ANG_LF_01	MobileNet	3.629	0.000	5.319	9.574	85.106	14.894	1.198
	7	WBG_ANG_LF_BK	DenseNet121	3.662	4.255	8.511	10.638	76.596	23.404	5.615
	8	WBG_ANG_LF_02	InceptionV3	3.663	4.255	7.447	11.702	76.596	23.404	3.777
	9	BGF_ANG_RT_BK	InceptionV3	3.673	4.255	5.319	12.766	77.660	22.340	3.422
	10	BGF_UPS_LF_01	VGG19	3.680	4.255	8.511	5.319	81.915	18.085	0.734
Avg D_a	1	WBG_ANG_RT_BK	MobileNet	0.205	31.915	59.574	8.511	0.000	100.00	1.161
	2	WBG_ANG_LF_BK	MobileNet	0.208	30.851	60.638	7.447	1.064	98.936	1.379
	3	WBG_ANG_RT_01	InceptionV3	0.212	36.170	57.447	5.319	1.064	98.936	3.450
	4	WBG_ANG_LF_01	DenseNet121	0.213	25.532	69.149	5.319	0.000	100.00	6.572
	5	BGF_ANG_RT_02	InceptionV3	0.219	27.660	68.085	3.191	1.064	98.936	3.646
	6	WBG_ANG_RT_02	InceptionV3	0.221	26.596	68.085	4.255	1.064	98.936	3.666
	7	WBG_ANG_LF_01	InceptionV3	0.221	38.298	54.255	6.383	1.064	98.936	4.330
	8	WBG_ANG_LF_BK	VGG16	0.222	32.979	57.447	8.511	1.064	98.936	0.506
	9	BGF_ANG_RT_02	MobileNet	0.223	37.234	52.128	9.574	1.064	98.936	0.978
	10	BGF_ANG_RT_BK	VGG16	0.225	28.723	62.766	8.511	0.000	100.00	0.758
Avg D_b	1	WBG_ANG_LF_01	MobileNet	0.694	9.574	31.915	32.979	25.532	74.468	0.992
	2	WBG_ANG_RT_02	MobileNet	0.726	11.702	28.723	32.979	26.596	73.404	0.783
	3	WBG_ANG_RT_BK	MobileNet	0.744	13.830	24.468	29.787	31.915	68.085	1.160
	4	WBG_ANG_RT_01	MobileNet	0.746	8.511	36.170	25.532	29.787	70.213	1.035
	5	BGF_ANG_RT_01	MobileNet	0.793	6.383	34.043	30.851	28.723	71.277	0.976
	6	WBG_ANG_RT_02	InceptionV3	0.799	6.383	27.660	38.298	27.660	72.340	3.577
	7	BGF_ANG_RT_02	MobileNet	0.810	8.511	31.915	32.979	26.596	73.404	0.956
	8	WBG_ANG_RT_01	InceptionV3	0.811	5.319	35.106	27.660	31.915	68.085	3.651
	9	BGF_ANG_RT_BK	InceptionV3	0.812	9.574	25.532	40.426	24.468	75.532	3.352
	10	BGF_ANG_LF_BK	InceptionV3	0.818	10.638	29.787	22.340	37.234	62.766	3.293

**Table 8:** Top 10 regression models with the lowest MAE values for Whiteness, Chroma, and Hue predictions.

Parameter	Rank	Data Set	Algorithm	MAE	Response Time (s)
Avg V_Whiteness	1	BGF_ANG_LF_02	MobileNet	3.160	0.964
	2	WBG_ANG_LF_01	DenseNet121	3.200	5.922
	3	BGF_ANG_RT_02	MobileNet	3.278	0.995
	4	BGF_UPS_LF_01	VGG16	3.287	0.799
	5	BGF_ANG_RT_BK	DenseNet121	3.314	7.921
	6	BGF_ANG_LF_BK	ResNet50	3.317	2.904
	7	WBG_ANG_RT_BK	ResNet50	3.329	2.765
	8	WBG_UPS_LF_01	VGG16	3.331	0.522
	9	WBG_ANG_RT_01	DenseNet121	3.338	4.995
	10	WBG_ANG_RT_01	ResNet50	3.356	3.253
Avg V_Croma	1	WBG_ANG_RT_BK	MobileNet	1.385	0.976
	2	WBG_ANG_LF_02	MobileNet	1.386	1.210
	3	WBG_ANG_LF_01	MobileNet	1.435	0.964
	4	WBG_ANG_RT_01	MobileNet	1.454	0.993
	5	WBG_ANG_RT_02	MobileNet	1.496	0.837
	6	BGF_ANG_LF_02	MobileNet	1.569	0.970
	7	BGF_ANG_RT_01	MobileNet	1.571	1.014
	8	BGF_ANG_RT_02	InceptionV3	1.574	3.326
	9	WBG_ANG_LF_BK	MobileNet	1.582	0.972
	10	WBG_ANG_LF_01	InceptionV3	1.590	3.573
Avg V_Hue	1	WBG_ANG_RT_BK	MobileNet	0.992	1.294
	2	WBG_ANG_LF_BK	MobileNet	1.034	0.980
	3	WBG_ANG_LF_BK	InceptionV3	1.053	3.418
	4	BGF_UPS_LF_01	MobileNet	1.098	1.183
	5	WBG_ANG_LF_01	MobileNet	1.108	0.960
	6	BGF_ANG_RT_02	DenseNet121	1.122	4.870
	7	WBG_ANG_RT_01	InceptionV3	1.124	3.397
	8	BGF_ANG_RT_02	MobileNet	1.125	1.170
	9	WBG_ANG_RT_02	MobileNet	1.126	0.886
	10	WBG_ANG_LF_02	DenseNet121	1.127	5.735
Avg D_Whiteness	1	BGF_ANG_LF_02	MobileNet	3.258	0.950
	2	BGF_UPS_RT_01	MobileNet	3.426	1.058
	3	WBG_ANG_LF_02	MobileNet	3.458	1.019
	4	BGF_ANG_RT_BK	MobileNet	3.458	0.827
	5	WBG_ANG_LF_01	DenseNet121	3.510	5.962
	6	BGF_ANG_LF_01	MobileNet	3.516	1.242
	7	WBG_ANG_RT_01	InceptionV3	3.516	3.346
	8	BGF_ANG_RT_02	MobileNet	3.521	0.890
	9	WBG_ANG_RT_01	DenseNet121	3.545	4.984
	10	WBG_ANG_RT_01	MobileNet	3.562	0.995
Avg D_Croma	1	BGF_ANG_RT_BK	MobileNet	0.443	0.922
	2	WBG_ANG_LF_BK	VGG16	0.456	0.461
	3	WBG_ANG_RT_BK	DenseNet121	0.464	4.986
	4	WBG_ANG_RT_BK	VGG19	0.466	0.659
	5	BGF_UPS_LF_01	MobileNet	0.466	0.983
	6	WBG_ANG_LF_02	MobileNet	0.467	0.985
	7	WBG_ANG_RT_BK	VGG16	0.469	0.386
	8	WBG_ANG_LF_BK	VGG19	0.473	0.655
	9	WBG_ANG_LF_02	DenseNet121	0.474	5.614
	10	WBG_UPS_LF_01	InceptionV3	0.475	3.282
Avg D_Hue	1	WBG_ANG_LF_02	MobileNet	0.708	1.018
	2	WBG_UPS_RT_01	DenseNet121	0.731	6.428
	3	WBG_ANG_LF_01	MobileNet	0.735	0.946
	4	BGF_ANG_RT_01	MobileNet	0.749	1.108
	5	WBG_UPS_RT_01	MobileNet	0.750	0.848
	6	BGF_ANG_LF_01	MobileNet	0.754	1.252
	7	WBG_ANG_LF_BK	MobileNet	0.762	1.001
	8	BGF_ANG_RT_01	DenseNet121	0.762	5.356
	9	BGF_ANG_LF_BK	MobileNet	0.762	0.943
	10	WBG_UPS_RT_01	InceptionV3	0.764	3.544

**Table 9:** Ranking of regression parameters based on MAE and their best-performing algorithms.

Rank	Parameter	MAE	MobileNet	VGG16
1	Avg D_a	0.205	✔	
2	Avg D_Croma	0.443	✔	
3	Avg D_b	0.694	✔	
4	Avg D_Hue	0.708	✔	
5	Avg V_a	0.805	✔	
6	Avg V_Hue	0.992	✔	
7	Avg V_b	1.011	✔	
8	Avg V_Croma	1.385	✔	
9	Avg V_L	2.849		✔
10	Avg V_Whiteness	3.160	✔	
11	Avg D_Whiteness	3.258	✔	
12	Avg D_L	3.513	✔	
Count of being best model			11	1

**Table 10:** Datasets associated with the best-performing regression models.

Parameters	Data Sets											
	WBG_ANG_RT_BK	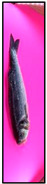	BGF_ANG_LF_02	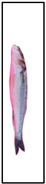	WBG_ANG_LF_01	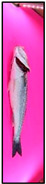	BGF_ANG_RT_BK	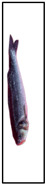	WBG_ANG_LF_02	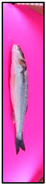	WBG_UPS_LF_01	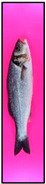
Avg D_a	✔											
Avg D_Croma							✔					
Avg D_Hue									✔			
Avg D_L			✔									
Avg D_Whiteness			✔									
Avg V_a	✔											
Avg V_b					✔							
Avg V_Croma	✔											
Avg V_Hue	✔											
Avg V_L											✔	
Avg V_Whiteness			✔									
Count of being best	4		3		2		1		1		1	

## Data Availability

The original contributions presented in this study are included in the article. The dataset used in this study was not obtained from any data repository; instead, it was created specifically for this research using sea bass (*Dicentrarchus labrax*) samples purchased for this purpose. The dataset has not been shared in any public data repository. Data are available from the corresponding author on reasonable request.

## Abbreviations

The following abbreviations are used in this manuscript:

AI	Artificial Intelligence
ANN	Artificial Neural Network
BeFAQT	Blockchain-enabled Fish Assessment and Quality Tracking System
CNN	Convolutional Neural Network
ESF	“Extended Seafood Freshness” dataset name
HSI	Hyperspectral Imaging
HSV	Hue, Saturation, Value
IoT	Internet of Things
K-NN	K-Nearest Neighbors
k-value	A chemical freshness indicator based on the proportion of ATP degradation products in fish.
LR	Logistic Regression
ML	Machine Learning
MSE	Mean Squared Error
NB-IoT	Narrowband Internet of Things
RF	Random Forest
RFR	Random Forest Regression
SC	Supply Chain
SFM	Sydney Fish Market
SVM	Support Vector Machine
SVR	Support Vector Regression
SWIR	Short-Wave Infrared
TBA	Thiobarbituric Acid
TVB-N	Total Volatile Basic Nitrogen
UV	Ultraviolet
VGG	Visual Geometry Group
VIS-NIR	Visible–Near-Infrared
VNIR	Visible and Near-Infrared
XGBoost	Extreme Gradient Boosting

## Appendix A

The Datasets used in this study are summarized in Table A1.

**Table A1:** Summary of the image datasets used in the study.

S. NO	Background	Angle No	Dataset Code	Sample Image
1	With background (WBG)	1	WBG_ANG_LF_01	
2		2	WBG_ANG_LF_02	
3		3	WBG_ANG_LF_BK	
4		4	WBG_ANG_RT_01	
5		5	WBG_ANG_RT_02	
6		6	WBG_ANG_RT_BK	
7		7	WBG_UPS_LF_01	
8		8	WBG_UPS_RT_01	
9	Background-free (BGF)	1	BGF_ANG_LF_01	
10		2	BGF_ANG_LF_02	
11		3	BGF_ANG_LF_BK	
12		4	BGF_ANG_RT_01	
13		5	BGF_ANG_RT_02	
14		6	BGF_ANG_RT_BK	
15		7	BGF_UPS_LF_01	
16		8	BGF_UPS_RT_01	

## Appendix B

Top 10 classification models with the highest accuracy scores for each categorical parameter are shown in Table A2.

**Table A2:** Top 10 classification models with the highest accuracy scores for each categorical parameter.

Parameter	Rank	Datasets	Algorithm	Accuracy	Precision	Recall	F1_Score	Response Time (s)
Skin Glossiness	1	WBG_ANG_RT_02	MobileNet	0.894	0.903	0.902	0.902	0.89
	2	WBG_ANG_RT_01	MobileNet	0.851	0.853	0.844	0.844	0.97
	3	WBG_ANG_RT_BK	DenseNet121	0.840	0.837	0.856	0.844	5.10
	4	WBG_ANG_LF_01	MobileNet	0.840	0.845	0.856	0.850	1.22
	5	WBG_ANG_LF_02	MobileNet	0.840	0.850	0.843	0.844	1.01
	6	BGF_ANG_RT_01	DenseNet121	0.830	0.831	0.853	0.830	4.78
	7	WBG_UPS_RT_01	DenseNet121	0.819	0.824	0.817	0.820	5.18
	8	BGF_UPS_LF_01	MobileNet	0.819	0.855	0.778	0.793	1.03
	9	BGF_UPS_RT_01	MobileNet	0.819	0.848	0.804	0.819	1.00
	10	WBG_ANG_LF_BK	DenseNet121	0.809	0.823	0.807	0.806	6.01
Skin Mucus	1	BGF_ANG_LF_01	EfficientNetB0	1.000	1.000	1.000	1.000	2.01
	2	WBG_ANG_LF_01	EfficientNetB0	1.000	1.000	1.000	1.000	2.93
	3	BGF_ANG_LF_02	EfficientNetB0	1.000	1.000	1.000	1.000	3.70
	4	WBG_ANG_LF_02	EfficientNetB0	1.000	1.000	1.000	1.000	1.64
	5	BGF_ANG_LF_BK	EfficientNetB0	1.000	1.000	1.000	1.000	3.17
	6	WBG_ANG_LF_BK	EfficientNetB0	1.000	1.000	1.000	1.000	1.74
	7	BGF_ANG_RT_01	EfficientNetB0	1.000	1.000	1.000	1.000	2.67
	8	WBG_ANG_RT_01	EfficientNetB0	1.000	1.000	1.000	1.000	2.93
	9	BGF_ANG_RT_02	EfficientNetB0	1.000	1.000	1.000	1.000	2.38
	10	WBG_ANG_RT_02	EfficientNetB0	1.000	1.000	1.000	1.000	2.26
Scale Arrangement	1	BGF_ANG_LF_01	EfficientNetB0	1.000	1.000	1.000	1.000	3.50
	2	WBG_ANG_LF_01	EfficientNetB0	1.000	1.000	1.000	1.000	2.97
	3	BGF_ANG_LF_02	EfficientNetB0	1.000	1.000	1.000	1.000	2.46
	4	WBG_ANG_LF_02	EfficientNetB0	1.000	1.000	1.000	1.000	3.18
	5	BGF_ANG_LF_BK	EfficientNetB0	1.000	1.000	1.000	1.000	2.27
	6	WBG_ANG_LF_BK	EfficientNetB0	1.000	1.000	1.000	1.000	2.29
	7	BGF_ANG_RT_01	EfficientNetB0	1.000	1.000	1.000	1.000	3.09
	8	WBG_ANG_RT_01	EfficientNetB0	1.000	1.000	1.000	1.000	1.96
	9	BGF_ANG_RT_02	EfficientNetB0	1.000	1.000	1.000	1.000	2.63
	10	WBG_ANG_RT_02	EfficientNetB0	1.000	1.000	1.000	1.000	5.75
Skin Texture	1	WBG_ANG_RT_BK	DenseNet121	0.904	0.892	0.912	0.897	5.20
	2	WBG_ANG_RT_01	MobileNet	0.872	0.877	0.873	0.871	0.98
	3	WBG_ANG_RT_02	MobileNet	0.872	0.882	0.874	0.864	0.89
	4	WBG_ANG_RT_BK	VGG16	0.872	0.866	0.869	0.867	0.75
	5	BGF_ANG_RT_02	MobileNet	0.862	0.872	0.867	0.868	1.10
	6	BGF_ANG_RT_BK	DenseNet121	0.851	0.850	0.819	0.830	5.45
	7	WBG_ANG_LF_BK	InceptionV3	0.851	0.841	0.848	0.844	3.18
	8	BGF_ANG_RT_BK	InceptionV3	0.851	0.860	0.862	0.857	3.39
	9	WBG_ANG_RT_BK	MobileNet	0.851	0.835	0.853	0.835	0.81
	10	WBG_ANG_RT_02	VGG16	0.851	0.842	0.858	0.839	0.54
Skin Odor	1	BGF_ANG_LF_01	DenseNet121	0.777	0.751	0.746	0.736	13.23
	2	WBG_ANG_RT_BK	DenseNet121	0.777	0.839	0.747	0.738	4.72
	3	WBG_ANG_RT_02	MobileNet	0.766	0.753	0.746	0.746	0.90
	4	WBG_ANG_RT_BK	MobileNet	0.755	0.743	0.689	0.703	1.02
	5	WBG_ANG_LF_BK	DenseNet121	0.734	0.741	0.710	0.712	5.54
	6	WBG_ANG_LF_01	MobileNet	0.734	0.749	0.695	0.704	0.80
	7	BGF_ANG_RT_BK	MobileNet	0.734	0.690	0.663	0.670	0.86
	8	WBG_ANG_LF_01	DenseNet121	0.723	0.724	0.684	0.694	6.03
	9	WBG_ANG_LF_BK	MobileNet	0.723	0.747	0.676	0.667	0.98
	10	WBG_ANG_RT_01	MobileNet	0.723	0.779	0.666	0.656	0.99
Gill Color	1	WBG_ANG_RT_02	MobileNet	0.894	0.920	0.861	0.870	1.36
	2	WBG_ANG_RT_01	DenseNet121	0.883	0.892	0.853	0.857	4.85
	3	BGF_ANG_RT_02	MobileNet	0.883	0.874	0.886	0.877	0.99
	4	WBG_ANG_RT_02	DenseNet121	0.862	0.852	0.853	0.852	5.19
	5	BGF_ANG_RT_01	MobileNet	0.862	0.846	0.842	0.843	1.43
	6	BGF_ANG_RT_02	DenseNet121	0.851	0.864	0.814	0.814	5.34
	7	WBG_ANG_RT_BK	MobileNet	0.851	0.879	0.808	0.814	0.85
	8	BGF_UPS_LF_01	MobileNet	0.851	0.848	0.856	0.849	1.01
	9	WBG_ANG_LF_BK	DenseNet121	0.840	0.834	0.833	0.833	5.57
	10	WBG_ANG_LF_02	MobileNet	0.840	0.856	0.853	0.841	0.96
Gill Mucus	1	WBG_ANG_RT_BK	DenseNet121	0.872	0.861	0.860	0.860	4.64
	2	WBG_ANG_LF_02	MobileNet	0.872	0.867	0.860	0.862	0.93
	3	WBG_ANG_RT_01	MobileNet	0.862	0.850	0.849	0.849	0.99
	4	WBG_ANG_RT_02	MobileNet	0.862	0.850	0.849	0.849	0.91
	5	BGF_ANG_RT_02	VGG16	0.862	0.867	0.836	0.840	0.53
	6	WBG_ANG_LF_02	DenseNet121	0.851	0.841	0.842	0.841	5.28
	7	BGF_UPS_LF_01	DenseNet121	0.851	0.858	0.852	0.848	4.39
	8	WBG_ANG_RT_BK	InceptionV3	0.851	0.854	0.821	0.820	3.39
	9	BGF_ANG_LF_BK	MobileNet	0.851	0.841	0.842	0.841	1.00
	10	WBG_ANG_RT_02	DenseNet121	0.840	0.842	0.840	0.832	5.29
Gill Odor	1	BGF_ANG_RT_01	MobileNet	0.809	0.790	0.789	0.788	0.96
	2	WBG_ANG_RT_BK	MobileNet	0.809	0.834	0.810	0.801	1.04
	3	WBG_ANG_LF_01	DenseNet121	0.798	0.795	0.771	0.773	8.33
	4	WBG_ANG_RT_02	DenseNet121	0.798	0.806	0.767	0.763	5.06
	5	BGF_ANG_LF_BK	MobileNet	0.798	0.808	0.784	0.778	1.00
	6	WBG_ANG_LF_BK	DenseNet121	0.777	0.762	0.732	0.721	4.80
	7	WBG_ANG_RT_02	MobileNet	0.777	0.794	0.736	0.734	0.89
	8	WBG_ANG_LF_01	MobileNet	0.766	0.776	0.740	0.736	0.81
	9	BGF_ANG_RT_02	MobileNet	0.766	0.766	0.761	0.752	0.98
	10	WBG_UPS_RT_01	MobileNet	0.766	0.749	0.749	0.748	1.00
Total Score	1	WBG_ANG_LF_02	MobileNet	0.574	0.169	0.290	0.211	1.03
	2	BGF_ANG_RT_02	DenseNet121	0.543	0.238	0.266	0.239	5.05
	3	WBG_ANG_RT_BK	MobileNet	0.543	0.304	0.325	0.283	0.84
	4	WBG_ANG_RT_01	DenseNet121	0.532	0.256	0.265	0.243	5.28
	5	WBG_ANG_LF_BK	DenseNet121	0.521	0.165	0.246	0.195	4.95
	6	BGF_ANG_RT_01	DenseNet121	0.521	0.161	0.232	0.188	4.70
	7	WBG_ANG_RT_BK	InceptionV3	0.511	0.278	0.257	0.235	3.38
	8	BGF_UPS_RT_01	MobileNet	0.511	0.217	0.259	0.231	0.90
	9	WBG_UPS_RT_01	DenseNet121	0.500	0.162	0.219	0.180	5.22
	10	BGF_ANG_RT_BK	InceptionV3	0.500	0.233	0.272	0.239	3.40
Purchasability	1	WBG_ANG_RT_BK	DenseNet121	0.989	0.992	0.985	0.988	4.83
	2	WBG_ANG_LF_BK	DenseNet121	0.979	0.971	0.984	0.977	5.40
	3	WBG_ANG_RT_01	MobileNet	0.979	0.971	0.984	0.977	1.08
	4	BGF_ANG_RT_02	MobileNet	0.979	0.977	0.977	0.977	0.97
	5	WBG_ANG_RT_02	VGG16	0.979	0.977	0.977	0.977	0.56
	6	WBG_ANG_RT_02	DenseNet121	0.968	0.968	0.962	0.965	5.82
	7	WBG_ANG_LF_BK	InceptionV3	0.968	0.958	0.975	0.966	3.36
	8	WBG_ANG_RT_02	MobileNet	0.968	0.958	0.975	0.966	0.86
	9	WBG_ANG_LF_01	DenseNet121	0.957	0.969	0.939	0.952	8.29
	10	WBG_ANG_LF_02	MobileNet	0.957	0.946	0.967	0.954	1.01
